# Dopaminergic dysfunction in the 3xTg-AD mice model of Alzheimer’s disease

**DOI:** 10.1038/s41598-021-99025-1

**Published:** 2021-09-30

**Authors:** Yesica Gloria, Kelly Ceyzériat, Stergios Tsartsalis, Philippe Millet, Benjamin B. Tournier

**Affiliations:** 1 Department of Psychiatry, University Hospitals of Geneva, Avenue de la Roseraie, 64, 1206 Geneva, Switzerland; 2 Department of Psychiatry, University of Geneva, Geneva, Switzerland; 3 Division of Nuclear Medicine, Diagnostic Department, University Hospitals of Geneva, Geneva, Switzerland; 4 Division of Radiation Oncology, Department of Oncology, University Hospitals of Geneva, Geneva, Switzerland; 5 Department of Brain Sciences, Faculty of Medicine, Imperial College London, London, UK

**Keywords:** Alzheimer's disease, Astrocyte, Microglia

## Abstract

Alzheimer’s disease (AD) is characterized by amyloid (Aβ) protein aggregation and neurofibrillary tangles accumulation, accompanied by neuroinflammation. With all the therapeutic attempts targeting these biomarkers having been unsuccessful, the understanding of early mechanisms involved in the pathology is of paramount importance. Dopaminergic system involvement in AD has been suggested, particularly through the appearance of dopaminergic dysfunction-related neuropsychiatric symptoms and an overall worsening of cognitive and behavioral symptoms. In this study, we reported an early dopaminergic dysfunction in a mouse model presenting both amyloid and Tau pathology. 3xTg-AD mice showed an increase of postsynaptic D_2/3_R receptors density in the striatum and D_2/3_-autoreceptors in SN/VTA cell bodies. Functionally, a reduction of anxiety-like behavior, an increase in locomotor activity and D_2_R hyper-sensitivity to quinpirole stimulation have been observed. In addition, microglial cells in the striatum showed an early inflammatory response, suggesting its participation in dopaminergic alterations. These events are observed at an age when tau accumulation and Aβ deposits in the hippocampus are low. Thus, our results suggest that early dopaminergic dysfunction could have consequences in behavior and cognitive function, and may shed light on future therapeutic pathways of AD.

## Introduction

Alzheimer’s disease (AD) is characterized by the accumulation of extracellular deposits formed from beta-amyloid (Aβ) protein and by intracellular neurofibrillary tangles of the Tau protein. These neurochemical hallmarks are accompanied by an increase in neuroinflammation which results in reactivity of astrocytes and microglial cells. However, all the therapies targeting these different actors have not allowed the emergence of effective treatments^1^. As a consequence, a better understanding in the mechanisms involved in the early stages of the pathology seems fundamental.

Beyond these AD biomarkers, a dysfunction of the dopaminergic system has been suggested. In human, an accumulation of amyloid plaques is present in the midbrain (cell bodies region of dopamine neurons) as in the ventral and dorsal striatum, major projecting regions of dopamine neurons^2–4^. Study of dopaminergic transmission through the evaluation of dopamine D_2/3_ receptor (D_2/3_R) density is inconclusive with studies that have shown an increase, a decrease, or the absence of effects of AD on the dopaminergic system^5–11^. From a clinical point of view, it has been shown that AD patients present dopaminergic dysfunction-related symptoms such as Parkinson-like dysfunctions, including decreased ambulatory activity and apathy, whose frequency, severity and prevalence (from 12 to 92%) depend on the severity of the pathology^2,12–21^. It is therefore possible that dopaminergic deficits participate in the overall worsening of the clinical condition. One possible mechanism for such an effect could be the relationship of the dopaminergic system with neuroinflammation. Indeed, beyond AD, it has been shown that dopamine receptor activation could have pro- or anti-inflammatory consequences^22–24^. In human, however, it remains difficult to determine the onset of dopaminergic neurotransmission dysfunction. The advantage of animal studies is that neurochemical or behavioral impacts could be measured precisely even before the appearance of traditional AD markers, in order to assess the potential of this phenomenon to be used as a biomarker for early diagnosis or even as a therapeutic target. In this idea, cell death in the midbrain of neurons expressing the tyrosine hydroxylase (TH) a rate-limiting enzyme in dopamine biosynthesis, has been shown at early ages in the APP/PS1 mouse AD model^25^. At the early stage of the pathology, TgF344-AD rats, another animal model of AD, showed goal-directed behaviors impairement^26^. At an age where Tau deposits are absent and Aβ is still diffused, we have previously reported behavioral dopaminergic hyper-function in this model^27^. APP/PS1^28,29^, Tau^30^ and mixed triple transgenic (3xTg-AD, APP_SWE_, PS1_M146V_ and Tau_P301L_)^31–36^ animal models have been shown to develop alterations in locomotor activity and this could result from changes in DA activity. Thus, these first studies suggest the implication of dopaminergic dysfunction within the early stage of AD pathogenesis.

To verify the hypothesis according to which dopaminergic transmission is impacted in early AD, we characterized in the midbrain and the striatum the markers of dopaminergic system (density of D_2/3_R, TH and DAT, dopamine transporter), glial reactivity (astrocytes and microglia) and behavioral effects (locomotion and locomotor response to stimulation of D_2_R) in heterozygous 3xTg-AD mice at an age when pathological markers are weakly expressed (Aβ and Tau). The use of heterozygous mice is advantageous to distinguish the onset of pathological signs since they are less severely manifested, and the neuropathology develops slower than in homozygous mice^37–39^.

## Results

### Increases in postsynaptic D_2/3_R and microglial reactivity in the striatum of heterozygous 3xTg-AD mice

**Figure 1:**
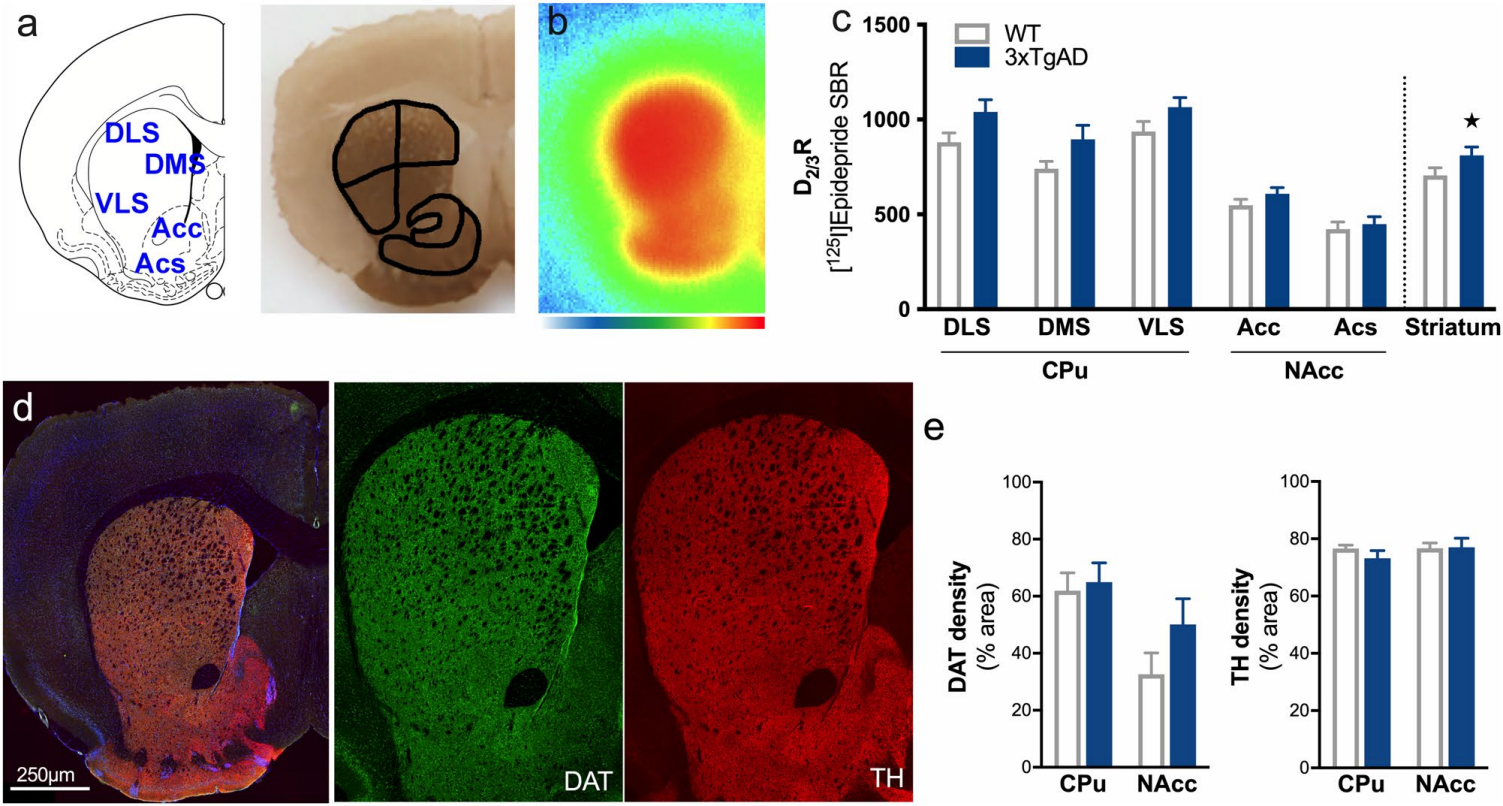
Increases in postsynaptic D_2/3_R in the striatum of 3xTg-AD mice. **(a)** Schematic representation of different areas of the striatum which have been drawn manually according to the acetylcholinesterase staining anatomy. **(b)** example of in situ labeling with [^125^I]Epidepride. Color scale refers to activity in the autoradiograms (from 0 to 500 MBq/mg). **(c)** Assessment of D_2/3_R density by [^125^I]Epidepride specific binding ratio (SBR) quantification. **(d)** Example of staining in the striatum for DAPI (blue), DAT (green) and TH (red). Left: merge image and right: images for DAT and TH. **(e)** Quantification of the area occupied by DAT and TH staining. ★: p < 0.05.

The quantification of D_2/3_R density in the striatum of 12-month-old 3xTg-AD mice compared to age-matched control animals revealed a global upregulation of the receptor expression which did not differ between the CPu (DLS, DMS, VLS) and the NAcc (Acc, Acs) (Fig. 1a–c, genotype: F_1,40_ = 11.65, p < 0.01; striatal subdivision: F_4,40_ = 49.95, p < 0.001; genotype × striatal subdivision: F_4,40_ = 0.74, p > 0.05). In contrast, the quantification of the area occupied by TH and DAT (dopamine transporter, involved in presynaptic dopamine reuptake) immunostaining showed no difference in the CPu (including DLS, DMS and VLS) and the VST (Acc and Acs) between control and 3xTg-AD mice (Fig. 1d,e, TH staining, genotype: F_1,12_ = 0.44, p > 0.05; striatal subdivision: F_1,12_ = 0.74, p > 0.05; genotype × striatal subdivision: F_1,12_ = 0.66, p > 0.05. DAT staining, genotype: F_1,12_ = 1.90, p > 0.05; striatal subdivision: F_1,12_ = 8.79, p < 0.05; genotype × striatal subdivision: F_1,12_ = 0.95, p > 0.05).

**Figure 2:**
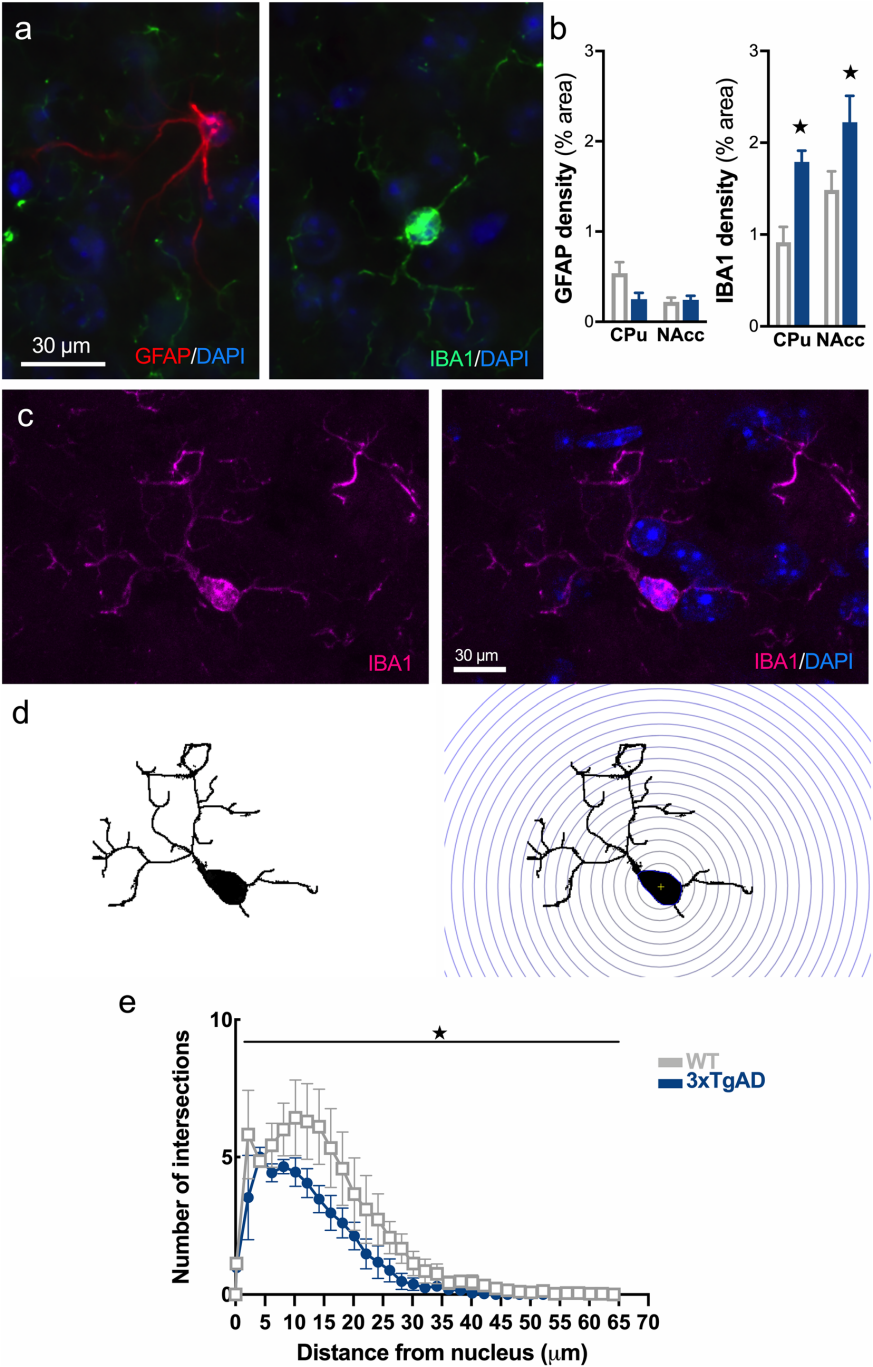
Early microglial reactivity in the striatum of 3xTg-AD mice. **(a)** Example of immunofluorescence for DAPI (blue), GFAP (red) and IBA1 (green) in the striatum. **(b)** Quantification of the area occupied by GFAP and IBA1 staining. **(c)** Representative example of IBA1^+^ microglial cells (magenta) and DAPI (blue) showing soma co-staining. **(d)** Procedure for isolating a microglial cell and applying the Sholl filter in concentric circles from the center of the soma to the end of ramifications. **(e)** Quantification of intersection number in WT (grey, open circles) and 3xTg-AD (blue, triangles) mice. ★: p < 0.05.

The evaluation of the inflammatory reaction with IBA1 and GFAP as markers of microglial and astrocytic reactivity, respectively, showed increases in the IBA1 immunoreactivity and no changes in GFAP immunoreactivity in the striatum (Fig. 2a,b, IBA1 staining, genotype: F_1,10_ = 15.7, p < 0.01; striatal subdivision: F_1,10_ = 6.06, p < 0.05; genotype × striatal subdivision: F_1,10_ = 0.11, p > 0.05. GFAP staining, genotype: F_1,12_ = 2.93, p > 0.05; striatal subdivision: F_1,12_ = 4.46, p > 0.05; genotype × striatal subdivision: F_1,12_ = 3.99, p > 0.05).

The complexity and functional state of microglia in the striatum was assessed on a cell-by-cell basis using Sholl analysis. For that, isolated IBA1^+^ cells were selected (Fig. 2c) and images were converted into binary images to remove the background (Fig. 2d, left panel). The number ramifications, number of processes and their length, calculated using concentric shell from the soma (Fig. 2d, right panel), reflect the cell complexity. As shown in Fig. 2e, the branching profile of microglia in 12-month-old 3xTg-AD is shifted to the left, demonstrating a reduction in intersection number (genotype: F_1,204_ = 29.1, p < 0.001; intersection number: F_33,204_ = 22.6, p < 0.001; genotype × intersection number: F_33,204_ = 1.00, p > 0.05), which is typical of microglial reactive state.

### Increases in presynaptic D_2/3_-autoreceptors in the midbrain of heterozygous 3xTg-AD mice, without modulation of inflammation markers

**Figure 3:**
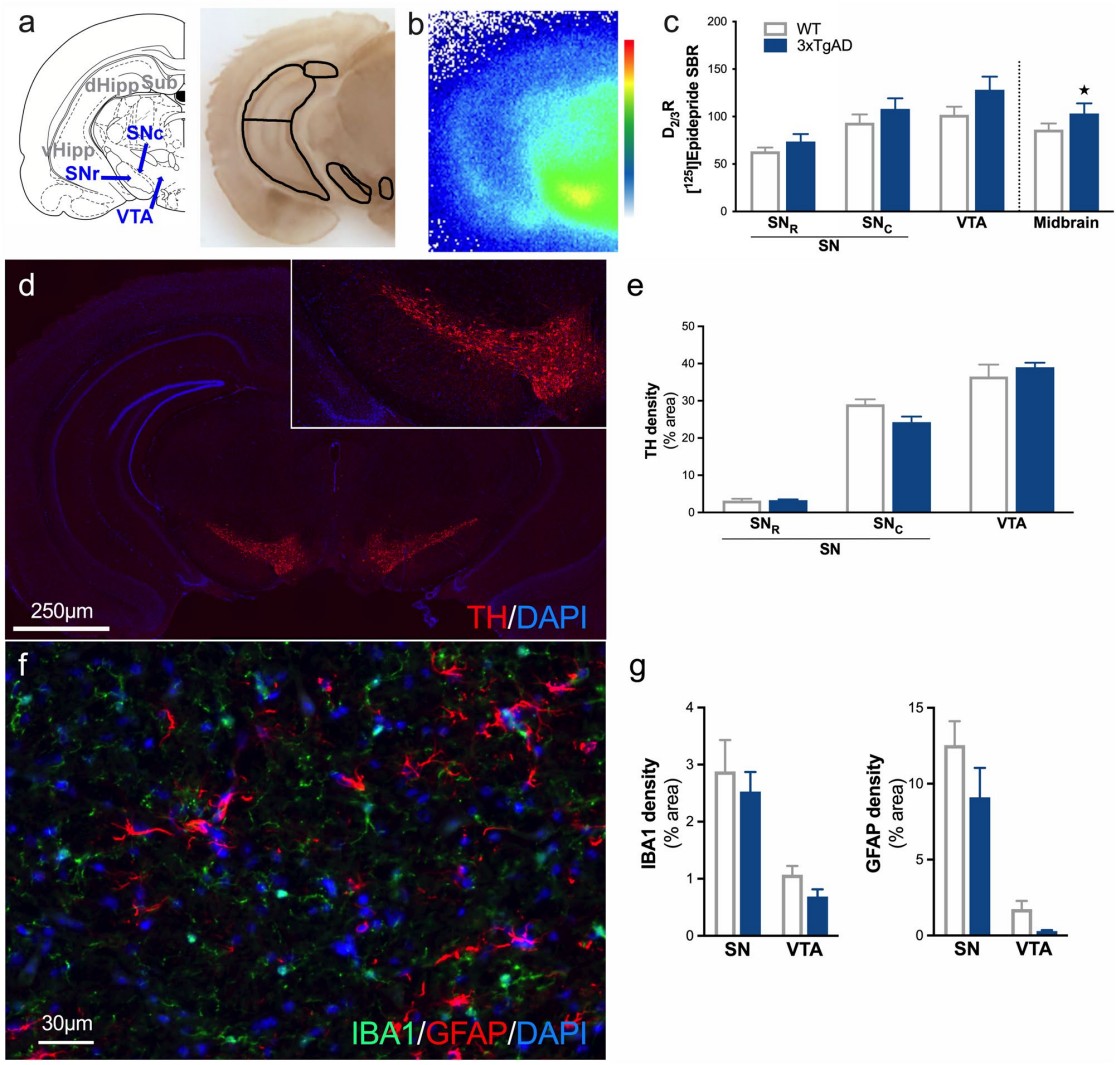
Increases in presynaptic D_2/3_R, without inflammation modulation, in the midbrain of 3xTg-AD mice. **(a)** Schematic representation of different areas of the midbrain and hippocampus which have been drawn manually according to the acetylcholinesterase staining anatomy. **(b)** Example of in situ labeling with [^125^I]Epidepride. Color scale refers to activity in the autoradiograms (from 0 to 500 MBq/mg). **(c)** Assessment of the D_2/3_R density by [^125^I]Epidepride specific binding ratio (SBR) quantification. **(d)** Example of staining at the level of the midbrain for DAPI (blue) and TH (red). **(e)** Quantification of the area occupied by TH staining. **(f)** Example of immunofluorescence for DAPI (blue), IBA1 (green) and GFAP (red) in the substantia nigra. **(g)** Quantification of the area occupied by IBA1 and GFAP staining. ★: p < 0.05.

In the midbrain, we also observed a global upregulation of D_2/3_R density in 12-month-old 3xTg-AD mice compared to WT mice (Fig. 3a–c, genotype: F_1,24_ = 4.83, p < 0.05; midbrain subdivision: F_2,24_ = 12.52, p < 0.001; genotype × midbrain subdivision: F_2,24_ = 0.38, p > 0.05). In contrast, quantification of the area occupied by TH immunostaining showed no difference between control and 3xTg-AD mice in the midbrain (Fig. 3d,e, genotype: F_1,18_ = 0.28, p > 0.05; midbrain subdivision: F_2,18_ = 226, p < 0.001; genotype × midbrain subdivision: F_2,18_ = 2.51, p > 0.05). IBA1 and GFAP immunoreactivity showed no changes in this region (Fig. 3f,g, IBA1 staining: genotype effect F_1,10_ = 1.0, p > 0.05; midbrain subdivision: F_1,10_ = 24.5, p < 0.001; genotype × striatal subdivision: F_1,10_ = 0.001, p > 0.05. GFAP staining: genotype effect F_1,11_ = 3.19, p > 0.05; midbrain subdivision: F_1,11_ = 50.9, p < 0.001; genotype × striatal subdivision: F_1,11_ = 0.52, p > 0.05).

### Sparse expression of Aβ and Tau markers in heterozygous 3xTg-AD mice

**Figure 4:**
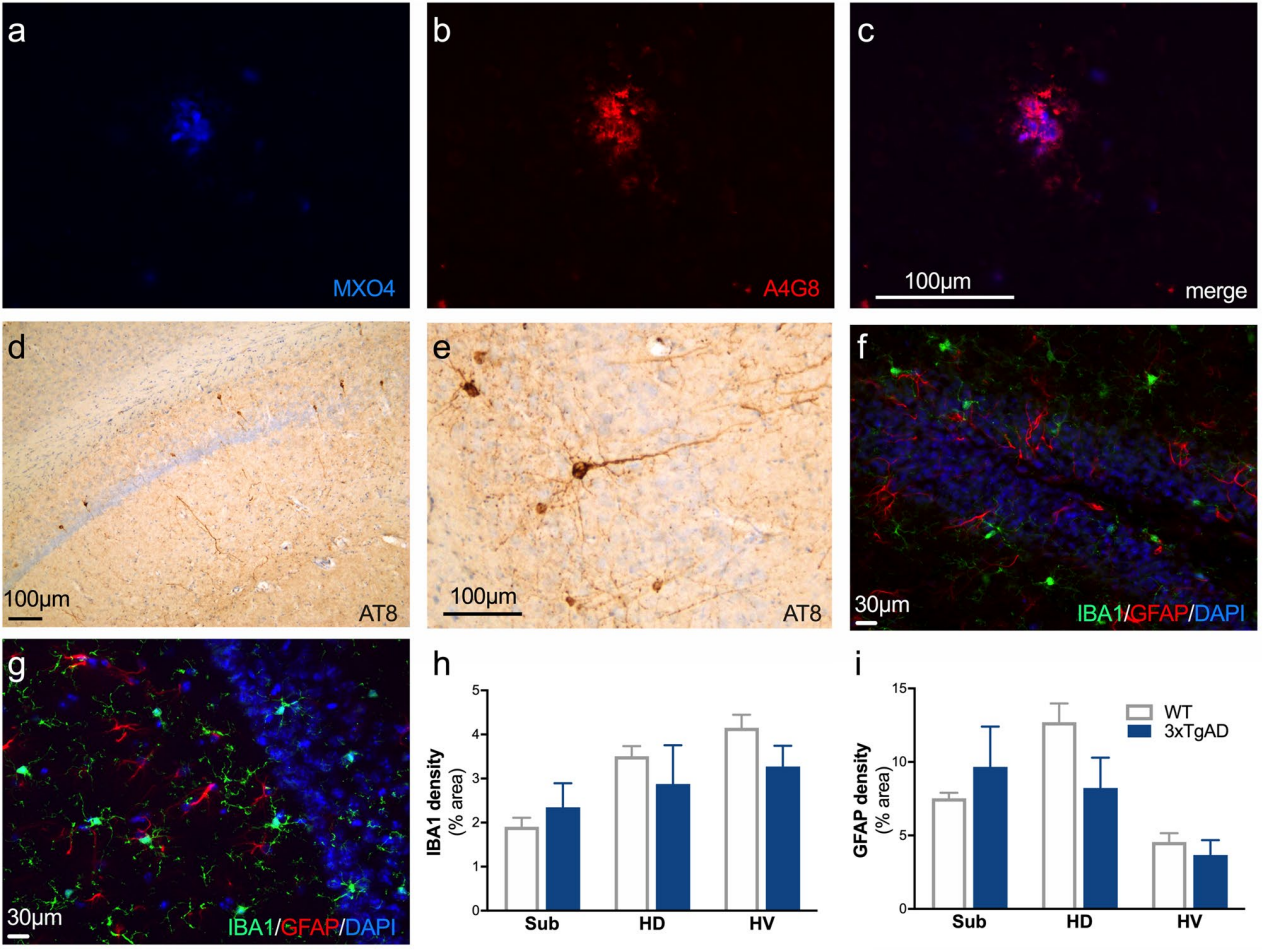
Sparse Aβ deposits restricted to the subiculum and low Tau levels in the hippocampus of 3xTg-AD mice. **(a–c)** A weak positive labeling of amyloid plaques with MXO_4_ (blue, **a**) and A4G8 (red, **b**) is observed and restricted to the subiculum. **(c)** Merge image of A and B. **(d)** A weak positive labeling for AT8 is observed only at the level of the hippocampus. **(e)** Example of positive AT8 cells showing positive cell bodies and neurites. **(f,g)** Example of immunofluorescence for DAPI (blue), IBA1 (green) and GFAP (red) in dentate gyrus **(f)** and dorsal hippocampus **(g)**. **(h,i)** Quantification of the area occupied by IBA1 **(h)** and GFAP **(i)** staining in hippocampal subdivisions.

MXO_4_ and A4G8 staining were sought in the regions of dopaminergic neurons (striatum, SN/VTA), cortex and hippocampus. The examination revealed the absence of extracellular Aβ deposits (i.e. MXO4^+^ or A4G8^+^ staining) in all the studied regions except for the few plaques present in the subiculum at 12 months old (Fig. 4a–c). The tau pathology was assessed by the use of AT8 immunostaining. No positive staining was observed in the striatum, midbrain and cortex. However, a few positive neurons were observed in the dorsal and ventral hippocampus (Fig. 4d,e). To reveal any inflammatory reaction in the hippocampus, IBA1 and GFAP staining were studied in the hippocampal subdivisions (Fig. 4f,g). No difference was observed between WT and 3xTg-AD mice (Fig. 4h,i).

### Behavioral alterations in 12-month-old heterozygous 3xTg-AD mice

**Figure 5:**
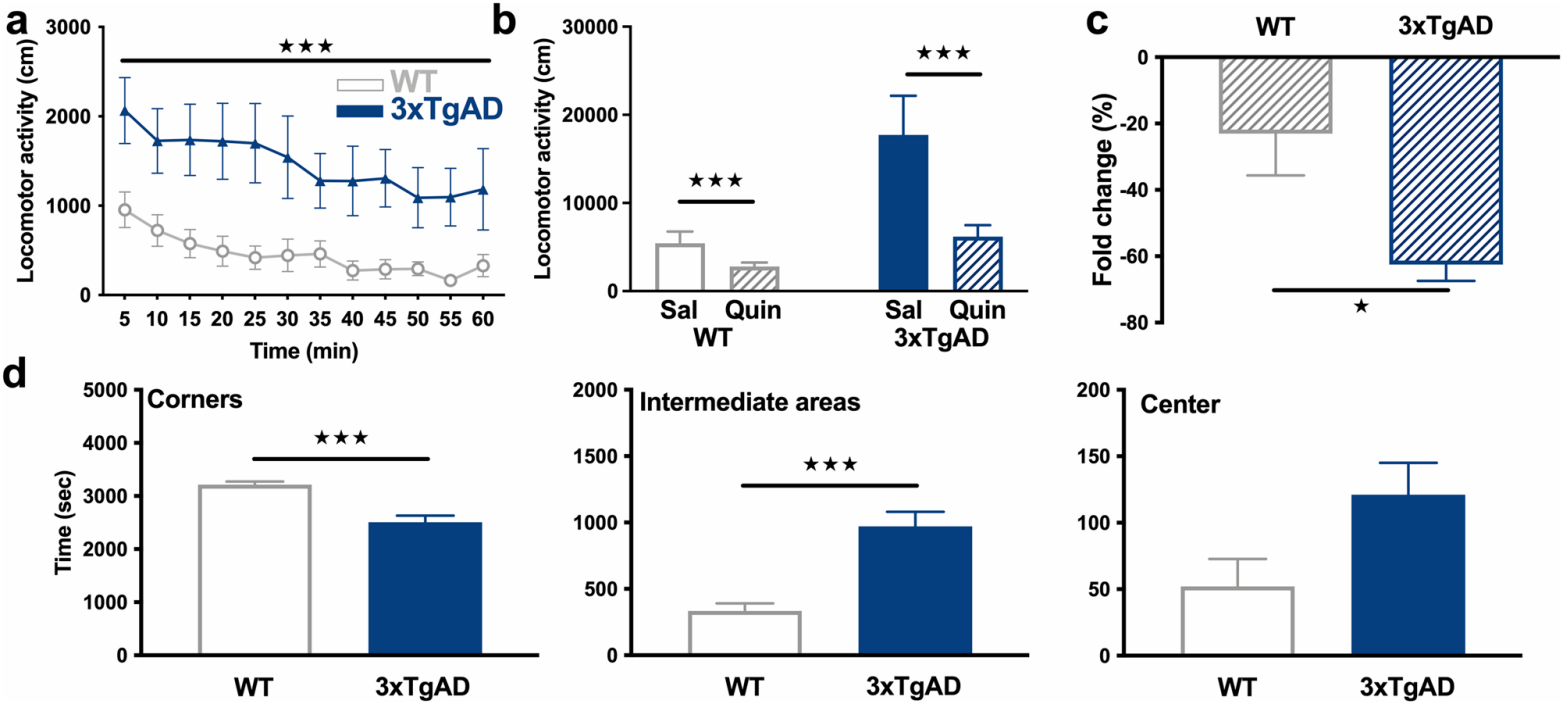
Behavioral alterations in 12-month-old 3xTg-AD mice. **(a)** Locomotor activity of WT (grey, open circles) and 3xTg-AD (blue, triangles) mice at baseline in 5 min blocks. **(b)** Total distance traveled, in response to saline (Sal) and quinpirole (Quin), a D_2_R agonist, in WT and 3xTg-AD mice. **(c)** Fold change in locomotion induced by quinpirole as compared to saline. **(d)** From left to right, time spent in corners, intermediate areas and central square of the open field during the baseline test in WT and 3xTg-AD mice. ★: p < 0.05, ★★: p < 0.01, ★★★: p < 0.001.

Two-way ANOVA indicated that 12-month-old hemizygous 3xTg-AD showed a higher spontaneous locomotor activity than WT mice, throughout the locomotor activity test (Fig. 5a, genotype: F_3,336_ = 87.4, p < 0.001; time: F_11,336_ = 3.53, p < 0.001; genotype × time: F_33,336_ = 0.40, p > 0.05).

Administration of the D_2_R preferring agonist quinpirole induced a reduction of locomotor activity as compared to the saline injection in both WT (p < 0.001) and 3xTg-AD (p < 0.001) mice (Fig. 5b). However, hemizygous 3xTg-AD mice showed a hyper-response to the inhibitory effect of D_2_R agonist (p < 0.05, Fig. 5c).

Estimation of anxiety levels by the analysis of the exploratory behavior of mice in the different areas of the open-field (the center being the most anxiogenic area) indicated that hemizygous 3xTg-AD mice were hypo-anxious with a reduction in the time past at the corners (p < 0.001), an increase in the time past in the intermediate squares of the open-field (p < 0.001) and a tendency to spend more time at the center (p = 0.0514, Fig. 5d).

### Dopamine-related locomotor dysfunctions are not present in 4-month-old heterozygous 3xTg-AD mice

To determine whether dopamine-related locomotor dysfunctions are already present at a younger age, a second cohort of 4-month-old animals performed the open-field test.

**Figure 6:**
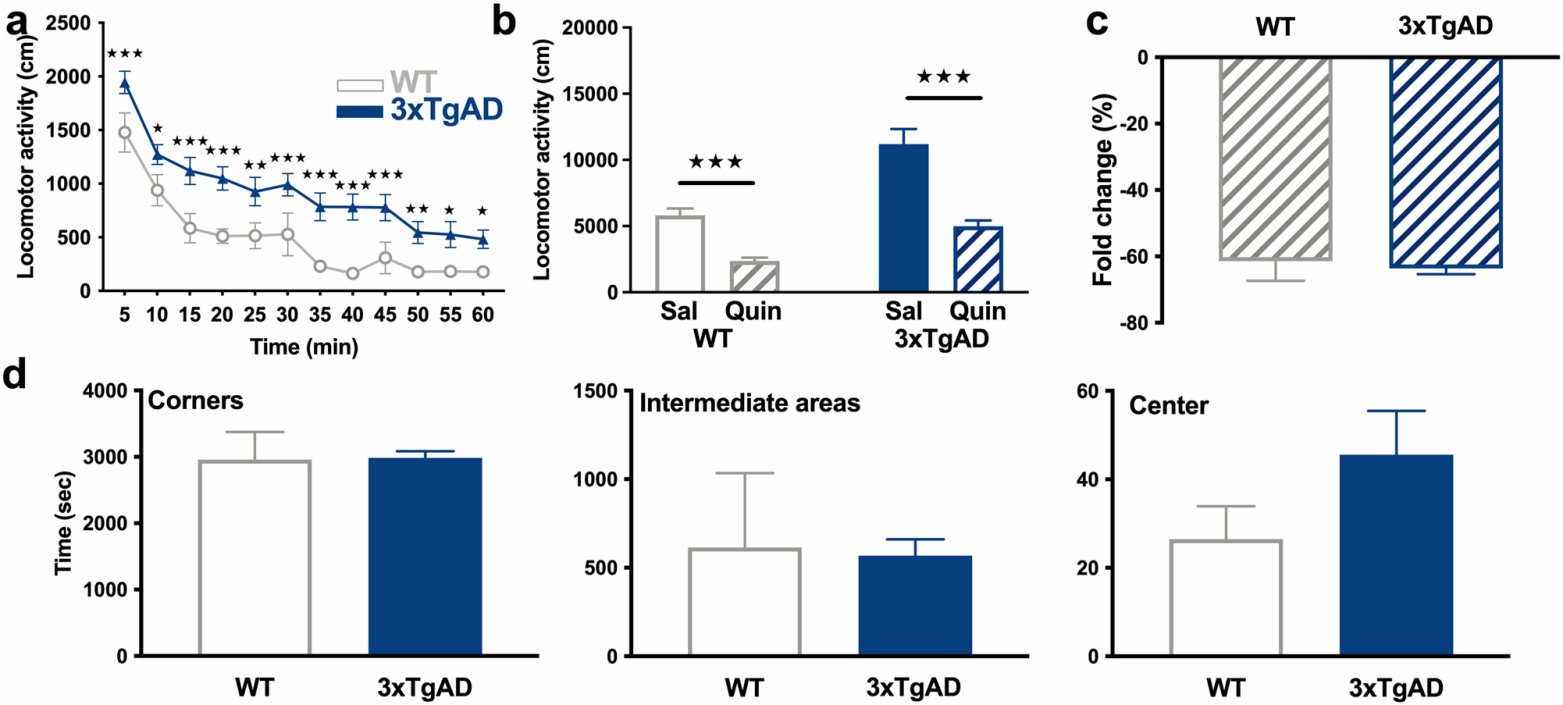
No dopaminergic-related behavioral alterations in 4-month-old 3xTg-AD mice. **(a)** Locomotor activity of WT (grey, open circles) and 3xTg-AD (blue, triangles) mice at baseline in 5 min blocks. **(b)** Total distance traveled, in response to saline (Sal) and quinpirole (Quin) in WT and 3xTg-AD mice. **(c)** Fold change in locomotion induced by quinpirole as compared to saline. **(d)** From left to right, time spent in corners, intermediate areas and central square of the open field during the baseline test in WT and 3xTg-AD mice. ★★★: p < 0.001.

Spontaneous locomotor activity of WT and hemizygous 3xTg-AD mice aged of 4 months was recorded in the open-field test. Two-way ANOVA indicated a higher time-dependent locomotor activity in 3xTg-AD mice (genotype: F_3,336_ = 129, p < 0.001; time: F_11,336_ = 32.15, p < 0.001; genotype × time: F_33,336_ = 3.34, p < 0.001). Post hoc test indicated that differences between hemizygous 3xTg-AD mice and WT concerned the entire 60-min time period, with different levels of significance (Fig. 6a), showing a hyperlocomotion at this age.

However, a significant inhibitory effect of quinpirole was observed in both WT (p < 0.001) and 3xTg-AD (p < 0.001) mice (Fig. 6b) and was not dependent of the genotype (p > 0.05, Fig. 6c). Consequently, the hyper-response to the inhibitory effect of D_2_R agonist is not yet present at 4 months of age.

The time past in the centre, corners and intermediate virtual squares of the open-field did not differ between hemizygous 3xTg-AD and control mice, indicating similar anxiety levels (p > 0.05, Fig. 6d).

## Discussion

Our study confirms and extends previous reports that show dysfunctions in dopaminergic system in a mouse model presenting both amyloid and Tau pathology. Our data demonstrated that 3xTg-AD mice show dopaminergic system alterations that occurs from the early stages of the pathology, marked by a low presence of intraneuronal Tau in the hippocampus and the presence of a limited number of Aβ deposits restricted to the subiculum. Regarding the dopaminergic system, we reported an increase in the density of postsynaptic D_2/3_R receptors in the striatum and in D_2/3_-autoreceptors of SN/VTA cell bodies. Functionally, there is an increase in locomotor activity at baseline and a D_2_R hyper-sensitivity to quinpirole stimulation. In the striatum, 3xTg-AD mice also show an increase in microglial cell density, which is not observed in either the hippocampus or the midbrain. This effect is accompanied at the cellular level by a reduction in complexity of the microglial cell morphology. These changes of microglial morphology and density reflect an early inflammatory response in the striatum, which could participate in altering dopaminergic functions. Finally, a reduction of anxiety-like behavior was also observed in the open-field test.

The involvement of dopaminergic system in Alzheimer's disease has been suggested in humans as well as in various animal models^2,18,25,40,41^. However, the precocious nature of these alterations remains poorly understood. In this study, the use of heterozygous mice makes it possible to obtain a slower evolution of the pathology^37–39^ and therefore to better observe the early phenomena, related to the age of the animals. At 12 months, a stage when mice show low amyloid and Tau accumulation in the hippocampus, we observed a general increase in D_2/3_R levels in the striatum and in the cell body regions of dopamine neurons. In humans, there is no consensus on such an alteration in D_2/3_R density^5–11^. However, these studies were carried out in patients whose pathological stage is, in comparison with our model, more advanced. As a decrease in dopamine synthesis in AD human brain has been described^42,43^ the increase in D_2/3_R density could be due to a compensatory phenomenon in response to this decrease in dopamine availability. Interestingly, such a compensation phenomenon has been observed in Parkinson's disease in unmedicated patients^44^, and in an experimental model of reduction in dopamine system activity^45,46^.

In the striatum, we observed an increase in D_2/3_R which could be linked with the hyper-locomotion of mice at baseline. In D_1_R and D_2_R knockout mice^47^, a decrease in locomotor activity was observed, which also corroborates the locomotor activity changes with the modulation of dopamine receptors expression. In addition, an increase in inflammation was observed through microglial reactivity. Indeed, microglial cells density is increased while their complexity is decreased, which reflects proliferation and activation of these cells. Importantly, microglial reactivity in the striatum appears to occur very early since within the hippocampus, one of the first region to develop amyloid plaques, astrocytes and microglial cells are not yet altered at this early stage. This further suggests that the glial cell alterations in brain regions associated to the dopaminergic system may play a role in the DA dysfunctions. Reciprocal links between dopaminergic function and the presence of inflammation have been suggested in some other studies. For example, stimulation of D_1_ and D_2_ dopamine receptors induces anti-inflammatory effects while stimulation of D_3_ and D_5_ dopamine receptors promotes inflammation^22,23^. In turn, inflammation-mediated effects appear to be amplified by the stimulation of D_3_R^24^. Not only expressed by neurons, dopamine receptors are also present on microglial cells and astrocytes, in animals as well as in humans^48–51^, which implies direct control of glial cell activity by dopamine. Thus, it is possible that the increase in D_2/3_R also affects glial receptors, which could participate in the modulation of inflammation. Overall, microglial activation could participate in a disorganization of the dopaminergic system.

At the level of the dopamine neurons cell bodies, D_2/3_-autoreceptors play a role in inhibiting dopamine neurons and therefore locomotion^52^. In response to an injection of the preferential D_2_R agonist, locomotor activity decreases, reflecting the activation of D_2/3_-autoreceptors. Twelve-month-old 3xTg-AD mice show increased inhibition of the locomotor activity, which may be correlated with neurochemical increases in D_2/3_-autoreceptors. This increase in D_2/3_-autoreceptors density could result in a stronger tonic inhibition of dopamine neurons. This idea is consistent with the decrease in the response of dopaminergic neurons to electrical and chemical stimulation^25,41,53^. However, the production and reuptake of dopamine by neurons does not still appear to be impacted in our 3xTg-AD mice, as shown by a stable level of TH and DAT, two key enzymes in dopamine level regulation. Interestingly, behavioral hyper-response to quinpirole is also observed early in a rat model of the pathology^41^. In another AD model, an increase in locomotion was also shown in the presence of constant dopamine levels compared to controls^28^. These observations validate the direct involvement of D_2/3_-autoreceptors in locomotion control, without necessarily a modulation of dopamine levels.

Beyond nigrostriatal and mesolimbic pathways, another pathway for activating striatal dopamine release should also be considered. In fact, neuronal activity in the hippocampus induces striatal dopamine release^54^. Demonstrations of hippocampal role in controlling locomotor activity have also been reported^55,56^. However, at the age of 12 months, our mouse model shows levels of Tau and amyloid in the hippocampus, even if relatively low, which could in themselves have pathological effects that cannot be totally excluded. It is therefore possible that amyloid and Tau accumulation in the hippocampus modify the transmission between the hippocampus and the striatum, resulting in a decrease of dopamine release which would generate an up-regulation of postsynaptic D_2/3_R. This hypothesis is consistent with a previous study demonstrating that homozygous 3xTg-AD mice display a decrease of cortical dopamine release at 10-month-old that was not present at 3-month-old. This decrease is accompanied by a loss of TH + neurons, not yet visible in our heterozygous mice^57^. It could be consequently interesting to repeat this study at an advanced stage, when mice present a more important amyloid and tau pathology to observe if dopaminergic deficits worsen with the progression of the pathology.

Finally, medium-spiny neurons of the striatum are also involved in the regulation of anxiety^58^. In the literature, 3xTg-AD females do not appear to show changes in anxiety at the light/dark and elevated plus maze tests^59,60^. However, increases in the time past at the center of the open-field was previously observed in females 3xTg-AD mice^61^. In our study, 12-month-old mice also showed a decrease in their anxiety level as shown by their exploratory behavior in the open field. Thus, it is therefore possible that the increase in D_2/3_R contributes to this reduction in anxiety.

In conclusion, basal hyperlocomotion is consistent with an increase in postsynaptic D_2_R and the inhibitory hyper-response to quinpirole consistent with an increase in D_2/3_-autoreceptors. Alterations in D_2/3_R signaling may represent early neuroadaptative changes at least in animal models of AD. These changes may contribute to increases in inflammation processes, to the development of AD pathology and, therefore, may represent an alternative strategy of earlier intervention. This idea is still speculative but cognitive improvements observed by stimulating treatment of dopaminergic system supports this hypothesis. Therefore, early deregulation of dopaminergic system could have consequences on the course of the pathology in terms of dysfunction of locomotion, anxiety, cognitive abilities, and goal-directed behaviors. All these phenomena are also observed in humans and suggest an early dysfunction of the dopaminergic system which may represent an interesting therapeutic target for AD patients. Future studies should address the long-term effects of therapy modifying D_2/3_R activity.

## Material and methods

### Animals

Female wild-type (C57B1/6 J-Sv129) and hemizygous 3xTg-AD (APPswe, Tau_P301L_ and PS1_M146V_^+/−^) mice aged 4 and 12 months were used. Animals were kept on 12 h light/dark cycle and provided with ad libitum access of food and water. All the experiments were approved by Ethics Committee for Animal Experimentation of the Canton of Geneva, Switzerland. All methods were performed in accordance with relevant guidelines and regulations and data are reported in accordance with Animal Research: Reporting In Vivo Experiments (ARRIVE) guidelines.

### Immunofluorescence and immunohistochemistry

Twelve-month-old 3xTg-AD and WT mice (n = 4/genotype) were transcardially perfused with 0.9% saline under 3% isoflurane anesthesia. Brains were removed and post-fixed in paraformaldehyde 4% (4 °C, 24 h), cryoprotected using sucrose gradient (5–20%, 4 °C, 48 h), and frozen in pre-cooled isopentane. Coronal section covering the rostro caudal extent on cryostat were cut and stored as free-floating slices (30 µm) in 1 × PBS 0.05% azide. For each region and each immunostaining, three slices separated by around 200 µm were used. Slices were mounted on gelatin-coated slides and, when require, immersed in the Methoxy-XO4 (MxO4) solution (30 min). Following two washes with 1 × PBS, slices were incubated overnight at 4 °C with the primary antibody in 1 × PBS, 1% BSA, 0.3% Triton X-100. Slices were washed twice in 1 × PBS, then incubated with the appropriated secondary antibody (when needed) for 60 min. After two washes in PBS, slices were exposed to Soudan Black (0.3% in 70% ethanol), then stained with DAPI (30 nM, when needed). When a DAB staining was required, a revelation with 0.2 mg/ml DAB (Sigma-Aldrich) in 1 × PBS containing 100 µl/L H_2_O_2_ was performed instead of Soudan black and DAPI staining. To stain amyloid deposits, slices were incubated with MxO_4_ (1/500, Tocris) at room temperature for 30 min. The primary and secondary antibodies were used as follows: mouse anti-GFAP-Cy3 (1/1000, Sigma), rabbit anti-Iba1 (1/300, Wako), mouse anti-phospho Tau (AT8, 1/500, Thermofisher), mouse anti-β amyloid 4G8 (1/500, Biolegend, San Diego), rabbit anti-TH (1/200, Thermofisher), rat anti-DAT (1/500, Thermofisher), mouse anti-rabbit HRP (1/100, Dako) and rabbit anti-mouse HRP (1/100, Dako), goat anti-rabbit Alexa 488-labeled (1/200, Invitrogen), goat anti-mouse Texas Red (1/200, Invitrogen), goat anti-rabbit Texas Red (1/200, Invitrogen), donkey anti-rat 488-labelled (1/200, Invitrogen). Images were acquired using a fluorescent microscope (Leica) or using the Zeiss Axioscan.Z1 scanner (Zeiss). Region of interest (ROI) was manually defined on the DAPI staining using the NIH ImageJ software. Since there is no clear anatomical landmark for SNc and VTA, ROI was determined according to the signal observed in the slices. The striatum was only divided in CPu and NAcc, due to the absence of good visualization of subregion delimitation. The mean grey value was measured to represent the average expression of the protein or immunoreactivity, and an intensity threshold was applied to measure the percentage area of immunopositive pixels, representing a density index of immunopositivity.

### Microglia morphology analysis

Morphometric analysis of IBA1^+^ cells from the striatum was performed using z-stack images taken with a confocal microscope (Zeiss, 3 shots per section, 3 sections per animal). The Sholl analysis was performed using the ImageJ software plugging. The soma of microglial cells was manually drawn, and concentric circles were automatically applied (from the soma center to the end of the microglial processes with a 2 μm distance between circles). The number of intersections along the Sholl radii and the area under the curves were analyzed as previously described^62^.

### In situ autoradiography

Twelve-month-old WT and 3xTg-AD mice (n = 5/genotype) were transcardially perfused with 0.9% saline under 3% isoflurane anesthesia, brains were removed and frozen in pre-cooled isopenthane. Serial coronal rostro caudal sections (30 µm) covering the striatum and the midbrain/hippocampus were cut on cryostat, mounted on gelatin-coated slides, and stored in − 20 °C. Slides were immersed in 1 × PBS (20 min), in the radioactive buffer (90 min), rinsed twice in 4 °C Tris-MgCl_2_ buffer (5 min) and briefly washed in cold water. The radioactive buffer consists of Tris-MgCl_2_ buffer (50 mM Tris HCl, 50 mM MgCl_2_) containing [^125^I]Epidepride alone or in presence of 10 µM of unlabeled ligands to determine the non-specific binding in the adjacent sections. Slides were air-dried then exposed with gamma-sensitive phosphor imaging plates (Fuji BAP-IS MS2325) for 24 h. To precisely define the anatomical landmarks, brain slices were treated with acetylcholinesterase staining. Autoradiograms were revealed using Fujifilm BAS-1800II phosphoimager in presence of homemade ^125^I calibration curves and analyzed using Aida Software V4.06 (Raytest Isotopenmessgerate GmbH), where the ROI were drawn manually. Based on an acetylcholinesterase staining to obtain good anatomical landmarks, the following ROIs were used: CPu (dorsolateral, ventrolateral, dorsomedian), NAcc (Shell and Core), SNc, SNr, VTA. For each ROI, the specific binding ratio (SBR) was calculated as follows: (radioactivity in ROI/radioactivity in ROI with 10 µM of unlabeled radiotracer) −1. For each animal and targets, 6–8 sections were analyzed and values in bilateral ROI were averaged.

### Locomotor activity

Four- and 12-month-old 3xTg-AD and WT mice (n = 8/group) performed a 3-day locomotor activity test to measure anxiety level, novelty habituation and the locomotor response to the injection of the D_2_R agonist quinpirole (0.5 mg/kg). The locomotion recording device consists of 4 square boxes of 45 × 45 × 40 cm virtually divided into 9 squares of identical size (15 × 15 cm). Three spatial areas were thus distinguished: the central square, the 4 corner squares and the 4 "intermediate" squares. A camera placed above allows to record the movements of the animals. The Noldus software allows an automatic analysis of the behaviors in terms of distance travelled and spatial location of the animal, per unit of time (5 min). Recordings of the animal’s behaviors was performed for 60 min for 2 consecutive days, 30 min after saline (day 1) or quinpirole (day 2) injection. On the day 1, an index of the level of anxiety was estimated and corresponds to the measurement of the time spent in the central, intermediate and corner squares. The novelty habituation to the open-field was also estimated on the day 1 by the difference of locomotion between the first and the last 15 min periods of the test. As mice are active mainly in the 0–45 min time period on day 1, the impact of quinpirole was calculated as follows: (distance travelled during the 0–45 min period on day 2 × 100/distance travelled during the 0–45 min period on day 1) – 100.

### Statistics

Two-way ANOVAs with the LSD *post-hoc* test was used to analyse locomotor behaviors (genotype × time), autoradiograms (genotype × ROI), immunofluorescence (genotype × ROI) and area under the curve (genotype × distance from soma). Comparison of anxiety, habituation levels and quinpirole effects were performed using a two-tailed t-test. Data are expressed as mean ± SEM.

## Data Availability

The datasets generated during and/or analyzed during the current study are available from the corresponding author on reasonable request.
